# Air Bubble Technique for Fundus Visualization during Vitrectomy in Aphakia

**DOI:** 10.1155/2017/4721540

**Published:** 2017-10-29

**Authors:** Mahmoud Mohamed Farouk, Takeshi Naito, Mohammed Elagouz, Hatem Ammar, Alahmady Hamad Alsmman, Engy Mohamed Mostafa

**Affiliations:** ^1^The Department of Ophthalmology, Sohag Faculty of Medicine, Sohag University, Sohag 82524, Egypt; ^2^The Department of Ophthalmology, Graduate School of Biomedical Sciences, Tokushima University, 3-18-15 Kuramoto-cho, Tokushima 770-8503, Japan

## Abstract

**Purpose:**

To evaluate the efficacy and safety of air bubble technique for vitrectomy in aphakia.

**Study Design:**

Prospective interventional uncontrolled case series.

**Methods:**

This study included 53 eyes of 53 patients who are phakic and indicated for phacovitrectomy (7 eyes, group 1), aphakic and indicated for vitrectomy (22 eyes, group 2), or underwent unplanned vitrectomy for immediate management of a phacoemulsification surgery complicated by rupture posterior capsule with dropped nucleus, fragments, or IOL (24 eyes, group 3). Cases with complicated vitreoretinal pathology were not included in this study. All vitrectomy surgeries were conducted by the air bubble technique in the anterior chamber. Main outcomes included anatomical success, visual acuity, and intraoperative and postoperative complications.

**Results:**

The surgical success was achieved in 50 eyes (94.3%). Conversion to BIOM viewing system was needed in the retinal detachment cases of groups 1 and 2. The mean overall LogMAR visual acuity was significantly improved from 1.29 ± 0.58 preoperatively to 0.56 ± 0.19 at the final visit, 6 months postoperatively (*P* < 0.001).

**Conclusion:**

The air bubble technique as visualization method for vitrectomy in aphakia is an effective and cheap technique for immediate management of complications of phacoemulsification surgery. This trial is registered with Pan African Clinical Trial Registry PACTR201709002466296.

## 1. Introduction

The vitrectomy viewing systems had been improved markedly in the last years. The current wide-angle viewing systems provided the surgeons with a panoramic view of the fundus with clear visualization even in air-filled eyes aiming to ensure good image of the peripheral fundus during surgery. The wide-angle viewing systems are divided into two types. One is the contact type which uses a contact lens and the other is the noncontact type. In the contact type, a contact lens is fixed on the cornea and the visibility abruptly worsens when the eye ball is tilted during surgery which is not a problem in the noncontact type. However, the corneal surface must be kept wet to maintain the visibility of the fundus [1–3].

The noncontact type enables a wider area of the fundus to be seen, but the quality of resolution is not sufficient for delicate manipulation. In contrast, the contact type is usually used to magnify the posterior pole for delicate maneuvers [4].

The cost of vitrectomy is an important issue to be considered. Scleral buckle procedure shows a modest cost savings over vitrectomy for repair of rhegmatogenous retinal detachment (RRD) [5].

Some hospitals cannot provide more than one viewing system in the ophthalmology operative theatre due to its high cost, a problem which pushed us to search for a cheap method for fundus visualization during vitrectomy. In 1989, Asfour and Nassar described a simplified technique for fundus visualization during vitrectomy in aphakia. They provided a clear view of the fundus during surgery simply by injecting a small air bubble that fills one-half to two-thirds of the anterior chamber [6].

The aim of this study is to evaluate the efficacy and safety of air bubble technique for fundus visualization during vitrectomy in aphakia.

## 2. Materials and Methods

### 2.1. Patients

This is a prospective, noncomparative study on patients who underwent 20-gauge pars plana vitrectomy (PPV) by air bubble technique from March 2012 to September 2014 at Sohag University Hospital.

According to the indication for PPV and the condition of the crystalline lens, the patients were divided into three groups. The phakic eyes which underwent combined phacoemulsification and PPV were considered as *group 1*, the aphakic eyes which underwent PPV only were considered as *group 2*, while those who underwent unplanned PPV for immediate management of a phacoemulsification surgery complicated by rupture posterior capsule with dropped nucleus, fragments, or IOL were considered as *group 3*. Cases with complicated vitreoretinal pathology were not included in this study; for example, advanced PVR and diabetic fibrovascular membranes.

All patients signed an informed consent form before intervention and ethical committee approval was obtained for this study. All patients were subjected to full medical and ophthalmic history taking and examination including best-corrected visual acuity (BCVA) measurement using Snellen's chart, intraocular pressure (IOP), anterior segment examination using slit lamp, and dilated fundus examination. Investigations (as needed) included ocular ultrasonography, optical coherence tomography, and fundus fluorescein angiography.

All operations were performed by the same surgeon (MF) using the 20-gauge transconjunctival cannula system (DORC, Zuidland, The Netherlands) and the Megatron S4 phacoemulsifier and vitrectomy system (Geuder, Heidelberg, Germany). All cases were designed to be performed by the air bubble technique, but a viewing system was prepared to be used if needed during surgery. Such viewing system was the binocular indirect ophthalmomicroscopy (BIOM 4) wide angle viewing system (OCULUS Optikgeräte GmbH, Wetzlar, Germany).

All patients underwent local monitored anesthesia care and received retrobulbar anesthesia. Further topical anesthesia was administered topically during surgery as needed. The periocular skin was prepared with 5% povidone iodine solution. The conjunctival sac was irrigated by povidone iodine solution and then irrigated by balanced salt solution (BSS). The eye was prepared and draped in a standard fashion, and a lid speculum was placed. Further surgical steps were variable according to the group.

### 2.2. Group 1

**Figure 1:**
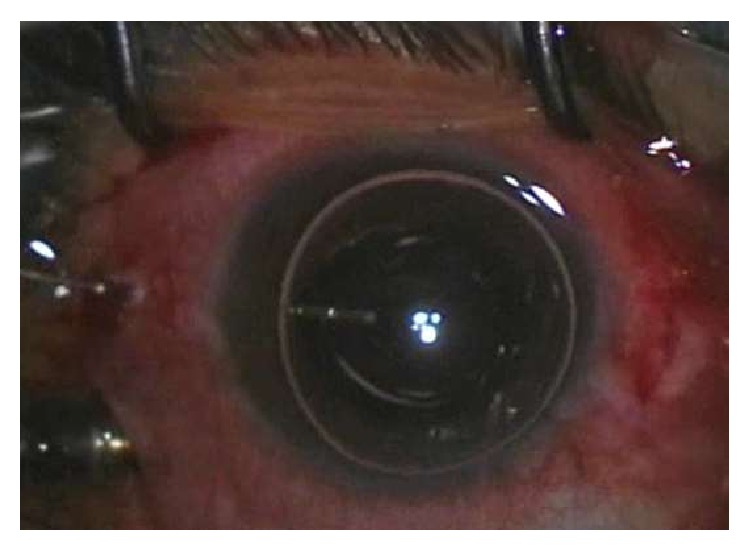
The anterior chamber (AC) partially filled by air bubble after completion of phacoemulsification to allow fundus visualization for vitrectomy.

In this group, all cases underwent phacoemulsification without implantation of IOL, which was postponed till the end of surgery. After completing the phacoemulsification, the anterior chamber (AC) was partially filled by air bubble (Figure 1) and the main corneal incision was closed temporary by single 10/0 Nylon suture. The three 20-gauge cannulas were inserted 3.5 mm from the corneoscleral limbus. The infusion catheter was connected to the inferotemporal cannula (which was the first to be inserted).

**Figure 2:**
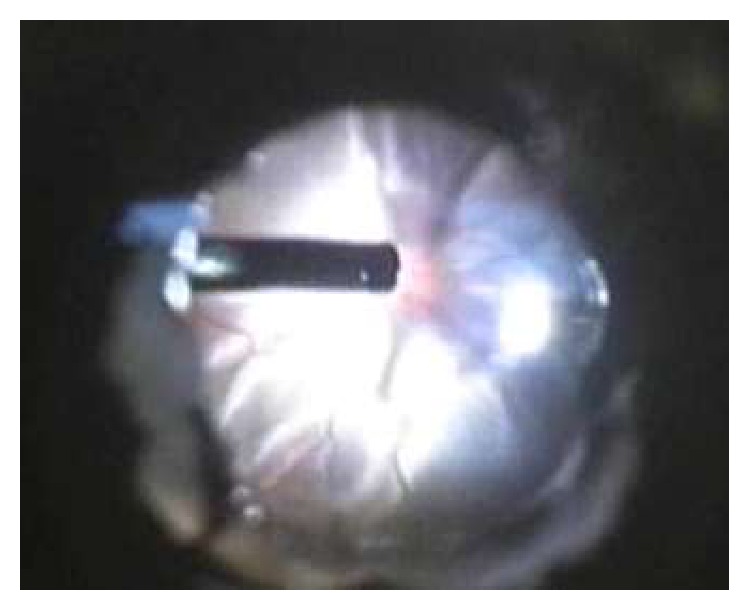
Fundus visualization during vitrectomy using the air bubble technique.

The vitrectomy procedure was performed according to the indication of each case. Fundus visualization during vitrectomy was achieved using the air bubble in the AC by adjusting the focus of the surgical microscope (Figure 2).

After completing the PPV procedure, the 10/0 Nylon suture was removed and the AC was filled with viscoelastic device instead of air. A foldable posterior chamber IOL was implanted through the corneal incision. The corneal wounds were sealed by stromal hydration, and the 3 sclerotomies were closed by 7/0 Vicryl sutures.

### 2.3. Group 2

In this group, all patients were already aphakic. The surgical procedure was the same as in group 1, but without phacoemulsification. The three 20-gauge cannulas were inserted at the beginning of surgery and the air bubble was injected directly into the AC through one of the two superior cannulas. This could be performed because all these cases had a defect in the posterior capsule.

### 2.4. Group 3

**Figure 3:**
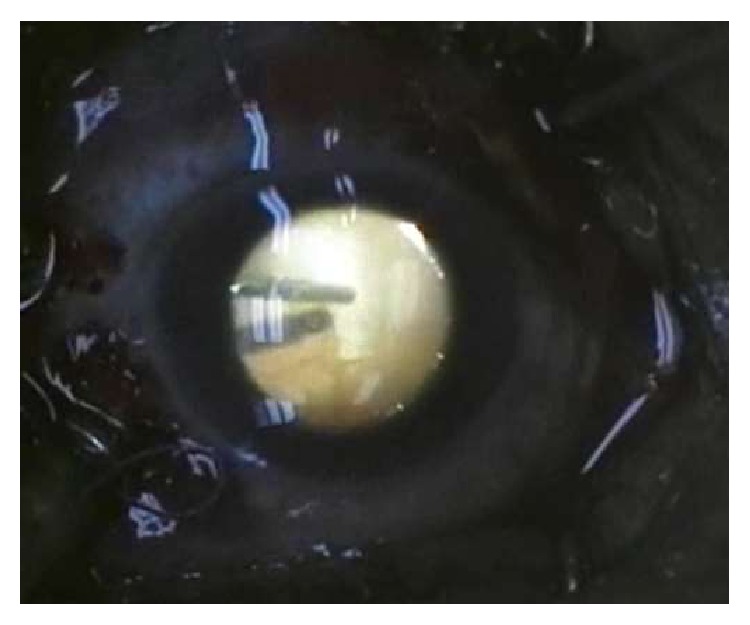
Vitrectomy performed by air bubble technique for immediate management of dropped lens fragment as a complication of phacoemulsification surgery.

In this group, the PPV was unplanned and the vitreoretinal surgeon was called for immediate management of a complication of phacoemulsification surgery (i.e., rupture posterior capsule with dropped nucleus, fragments, or IOL). The main corneal incision was closed temporary by single 10/0 Nylon suture. The procedure was completed as in group 1 (Figure 3).

**Figure 4:**
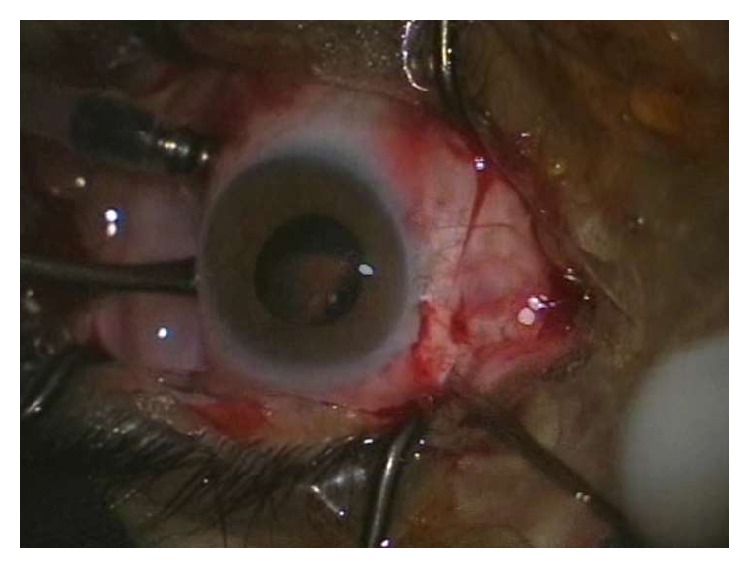
Peripheral vitrectomy performed by scleral depression and direct visualization of the peripheral retina without a viewing system.

In all groups, if the air bubble escaped from the AC through any incision, decreased in size, or fragmented into multiple bubbles, the procedure was stopped and reinjection of air bubble in the AC was performed. At the step of vitreous base shaving, the bubble was removed from the AC and peripheral vitrectomy was performed by scleral depression and direct visualization of the peripheral retina without a viewing system (Figure 4). Some cases could not be completed by this air bubble visualization technique and we had to shift to the BIOM system at certain steps.

At the end of surgery, topical antibiotic and steroid ointment was administered, and the eye was patched and shielded. Intraoperative complications and the methods of their management were recorded. Surgical success of the air bubble technique was defined as completing the whole steps of PPV procedure in a standard manner, without the need to shift to another viewing system.

### 2.5. Postoperatively

Patients were evaluated 1 day, 5 days, 1 month, 3 months, and 6 months after surgery. At each follow-up, the following data were recorded: best-corrected visual acuity, IOP, and findings of slit-lamp biomicroscopy of the anterior and posterior segments. All patients had at least 6 months follow-up. Main outcomes included surgical success, visual acuity, and intraoperative and postoperative complications.

### 2.6. Statistical Analysis

All analyses were performed using SPSS for Windows version 9.0 (SPSS Inc., Chicago, IL). Data were expressed as mean ± standard deviation (SD). A paired Student's *t*-test was used to make statistical comparisons between preoperative and postoperative LogMAR visual acuity and IOP. A *P* value < 0.05 was considered as significant.

## 3. Results

### 3.1. Baseline and Demographic Data

Fifty three eyes of 53 patients (29 male and 24 female) underwent PPV with the air bubble technique. The mean age was 56.9 ± 11.4 years (range 25–89 years). Tables 1, 2, and 3 summarize demographic and baseline preoperative data of each group.

### 3.2. Surgical Data

In group 1, fundus visualization was accepted by the air bubble in all cases, but some distortion was noticed at the periphery of the field of vision. On the other hand, we faced some events during surgery which made this visualization technique not helpful in certain situations. Irregular rupture of the posterior capsule occurred accidentally by the vitreous cutter in one case with RRD, resulting in distortion of the posterior surface of the air bubble with subsequent distortion of the view. This situation was managed by complete removal of the posterior capsule by the cutter to allow the injection of a regular air bubble. At the step of fluid-air exchange in the other case with RRD, the visualization was very difficult due to the presence of two air bubbles (one in the AC and one in the vitreous cavity). We had to shift to the BIOM system to complete the procedure. In the case of epiretinal membrane, we could peal the ERM successfully by the air bubble technique after increasing the microscope magnification.

In group 2, all operations could be completed by the air bubble technique. Cases with dropped IOL, nucleus, or lens fragments from previous phacoemulsification surgery were easily completed as well as the cases with posterior dislocation of crystalline lens (either traumatic or syndromatic). One case in this group had aphakic RD, in which we faced difficult visualization at the step of fluid-air exchange, so we completed the case by using the BIOM system.

In group 3, all operations were performed by the same surgical microscope which was used for the original phacoemulsification surgery and was not mounted by the BIOM system. There was no need to shift to another visualization system in any case.

No intraoperative complications related to the procedure were recorded in the three groups. Conversion to BIOM viewing system was needed in the RD cases of groups 1 and 2.

### 3.3. Visual Acuity Outcomes

The BCVA was measured using Snellen's chart and converted to LogMAR visual acuity. The mean overall LogMAR visual acuity was significantly improved from 1.29 ± 0.58 preoperatively to 0.56 ± 0.19 at the final visit, 6 months postoperatively (*P* < 0.001). There was also a significant improvement of visual acuity in each group separately. These results are summarized in Table 4.

### 3.4. Surgical Success

The overall surgical success of the air bubble technique was achieved in 50 (94.3%) eyes. In 3 eyes, we had to shift to the BIOM system.

## 4. Discussion

This study reports the results of a prospective analysis of the use of air bubble technique for fundus visualization during vitrectomy in aphakia. We have performed some operations using this technique in variable indications. The advantages of this technique were clear in immediate management of complications of phacoemulsification surgery. Usually, the microscope used for phacoemulsification surgery is not suitable for vitreoretinal surgery, because it is not mounted by visualization system as the BIOM system. So, this technique allows the immediate management of this situation by using the same microscope. Previous studies showed that early management of dropped nucleus or fragments carried a better prognosis and visual outcome with less complications than delayed vitrectomy [7, 8]. Another advantage is that the patient is managed by one operation without the need to go to the operative theatre again.

In other indications of PPV (i.e., RD and ERM). The only advantage of the air bubble technique was the decreased coast. But, on the other hand, our study found that the operative theatre must be equipped with a wide angle viewing system as BIOM to be a ready alternative to the air bubble technique. So, the surgeon cannot guarantee that he can complete the operation by the air bubble technique, specially at certain steps as fluid-air exchange or ILM pealing.

In conclusion, our study demonstrates that the air bubble technique as visualization method for vitrectomy in aphakia is an effective and cheap technique for immediate management of complications of phacoemulsification surgery. But, in other indications, it is much better to use a wide angle viewing system.

## Supplementary Material

The supplementary video shows the different steps of the air bubble technique for vitrectomy.

## Figures and Tables

**Table 1:** Demographic and base line preoperative data of group 1 (7 patients), who underwent combined phacovitrectomy.

Age (year), mean ± SD (range)	54.5 ± 7.7 years (41–64)	

Sex, number	Male	2
	Female	5

Surgical indication, number	Rhegmatogenous RD	2
	Diabetic vitreous hemorrhage	3
	Dense asteroid hyalosis	1
	Epiretinal membrane	1

SD: standard deviation; RD: retinal detachment.

**Table 2:** Demographic and baseline preoperative data of group 2 (22 patients), who were aphakic and underwent vitrectomy.

Age (year), mean ± SD (range)	53.8 ± 10.6 years (25–89)	

Sex, number	Male	14
	Female	8

Surgical indication, number	Dropped IOL from previous phacoemulsification surgery	8
	Dropped nucleus or lens fragments from previous phacoemulsification surgery	11
	Traumatic posterior dislocation of crystalline lens	1
	Rhegmatogenous RD	1
	Syndromatic posterior dislocation of crystalline lens (Marfan syndrome)	1

SD: standard deviation; IOL: intraocular lens; RD: retinal detachment.

**Table 3:** Demographic and base line preoperative data of group 3 (24 patients), who underwent unplanned vitrectomy for immediate management of a complication of phacoemulsification surgery (i.e., rupture posterior capsule with dropped nucleus, fragments, or IOL).

Age (year), mean ± SD (range)		

Sex, number	Male	13
	Female	11

Surgical indication, number	Dropped nucleus or lens fragments	21
	Dropped IOL	3

SD: standard deviation; IOL: intraocular lens.

**Table 4:** Preoperative and postoperative visual acuity results.

	Overall LogMAR Mean ± SD	LogMAR for each group Mean ± SD		
		Group 1	Group 2	Group 3
Preoperative	1.29 ± 0.58	2.13 ± 0.92	1.43 ± 0.40	0.91 ± 0.10
Postoperative (6 m)	0.56 ± 0.19 *P* < 0.001	0.68 ± 0.21 *P* < 0.001	0.68 ± 0.15 *P* < 0.001	0.42 ± 0.11 *P* < 0.001

LogMAR: logarithm of the minimum angle of resolution; SD: standard deviation.
